# Medical student and resident perceptions when working together in resident continuity clinics

**DOI:** 10.1080/10872981.2020.1827532

**Published:** 2020-10-05

**Authors:** Tina Chaalan, Deborah Landis Lewis, Kelly O’Connor, Bryan Popp, Maya Hammoud, Erika L Mowers

**Affiliations:** a OB/GYN PGY-4 Resident, St. Joseph Mercy Hospital, Ann Arbor, MI, USA; b Associate Residency Program Director OB/GYN, St. Joseph Mercy Hospital, Ann Arbor, MI, USA; c Residency Program Director OB/GYN, St. Joseph Mercy Hospital, Ann Arbor, MI, USA; d OB/GYN Department Chair, St. Joseph Mercy Hospital, Ann Arbor, MI, USA; e OB/GYN Medical Student Clerkship Director, Michigan Medicine, Ann Arbor, MI, USA; f OB/GYN Medical Student Clerkship Director, St. Joseph Mercy Hospital, Ann Arbor, MI, USA

**Keywords:** Medical student, teaching, resident education, resident training

## Abstract

**Background:**

Resident continuity clinics (RCCs), where residents see patients largely independently, is a common requirement for residency programs in the USA. Students often participate in these clinics but it is unknown how this effects resident learning or student satisfaction.

**Objective:**

This study aims to describe effects on the learning environment when students and residents work together in an RCC.

**Design:**

Separate surveys were administered to residents and students working at St. Joseph Mercy Hospital (SJMH) in Ann Arbor, Michigan, from 2016–2018.

**Results:**

Response rates were 79/116 (68.1%) for students and 21/24 (87.5%) for residents. A one-sample Wilcoxon signed rank test was used to test whether most five-level Likert-type scale responses were ‘agree’ or ‘strongly agree.’ Of medical students, 88.6% enjoyed working with residents (p < 0.001) with 60.8% indicating residents were effective teachers (p < 0.001). The majority of residents (85.7%) were neutral, agreed, or strongly agreed that they enjoyed working with students (p < 0.001). However, 61.9% of residents believed they were too busy to be effective teachers (p < 0.001).

**Conclusions:**

Both residents and students positively viewed their interactions in RCCs. Although most students felt residents were effective teachers, most residents worried about their ability to balance clinical care and teaching responsibilities.

## Introduction

Patient continuity is an Accreditation Council of Graduate Medical Education (ACGME) requirement for primary care residencies in the USA including Internal Medicine, Family Medicine, Pediatrics and OB/GYN. Most residencies fill this requirement through resident continuity clinics (RCCs) where residents serve as the primary providers with faculty supervision. An RCC is based on the traditional private practice model with the exception that its providers are resident physicians operating under the supervision of attending physicians. RCCs are extremely common in U.S primary care residencies. This is different than traditional clinics where learners serve as adjuncts to the attending physician. At the authors’ institution, St. Joseph Mercy Hospital (SJMH) in Ann Arbor, Michigan, students rotate exclusively in an RCC for their OB/GYN ambulatory experience. Seven students typically rotate on the OB/GYN service at any given time, including five medical students (M.D and D.O programs) and two physician assistant (PA) students. No more than two students are in clinic at any one time. Nevertheless, this results in a multitude of learners in one place.

Residents are expected to be the primary educators for these students but training for this is frequently minimal or nonexistent. This research was conducted to determine if students should be moved to traditional attending clinics to improve overall education and to explore perspectives of students and residents in regards to this specific learning environment.

Previous studies have shown that perceived quality of resident interactions and increased resident availability result in better clerkship satisfaction scores [1,2]. Specifically, students reported they are more comfortable asking questions with resident preceptors in comparison to attendings [3]. Residents frequently serve as role models and can greatly influence a student’s choice of specialty [4–6]. Objectively, students who report better quality resident instruction achieve higher scores on exams [7–9]. It remains unknown if students prefer to learn from residents or more experienced physicians in OB/GYN ambulatory clinics.

Balancing the demands of teaching and clinical care presents challenges even for experienced clinicians. In a 2017 study, 44% of community preceptors felt students had a negative impact on their work hours [10]. Residents share these concerns, and especially, junior level residents [11]. It is therefore questionable if the continuity clinic experience for residents is compromised by the presence of students. However, despite the impression that students may inhibit clinical efficiency, objective data shows equivalent or even increased clinical productivity when medical students are present [12,13].

Our objectives were to assess resident and student perceptions of their learning experience when working together in an RCC.

## Methods

The authors report no conflict of interest or financial disclosures. Before data collection, this study was given exempt status by the SJMH Institutional Review Board. All data storage and handling maintained compliance with SJMH and Trinity Health policies.

Two surveys were created using Survey Monkey and distributed via email [14]. The survey links were distributed a total of five times, approximately every week starting July 3^rd^, 2018 and ending July 26^th^, 2018. Each time the link was sent, it served as a reminder. Duplicate submissions from the same participant were prohibited.

Participants were students and residents who had rotated in the OB/GYN RCC at SJMH between January 1^st^, 2016 and January 31^st^, 2018. The survey questions were formatted with a five-point Likert-type scale with responses 1 = ‘strongly disagree,’ 2 = ‘disagree,’ 3 = ‘neutral,’ 4 = ‘agree,’ and 5 = ‘strongly agree.’ Student surveys were composed of fourteen questions in total. Seven questions assessed the students’ general clinical experience working with residents and seven questions were specific to their interactions in the OB/GYN RCC at SJMH. Resident surveys were composed of ten questions inquiring about their experience with students in their continuity clinic.

Statistical analysis was performed using Microsoft Excel 2008 and R version 3.5 [15]. A one-sample Wilcoxon signed rank test was used to test the authors’ hypothesis that most responses would be ‘agree’ or ‘strongly agree’ (i.e., greater than ‘neutral’). Differences in survey responses between demographics subgroups were assessed using Wilcoxon-Mann-Whitney tests. A P value of less than 0.05 was considered statistically significant.

## Results

### Student survey

A total of 116 surveys were sent out, and 79 students (68.1%) responded. As shown in Table 1, the majority of students agreed or strongly agreed that they generally enjoyed working with residents (88.6% vs. 11.4%, P < 0.001) and residents had enough knowledge to be effective teachers (86.1% vs. 13.9%, P < 0.001). Specific to OB/GYN RCC, students agreed or strongly agreed that residents enhanced their learning (68.4% vs. 31.6%, P < 0.001), were able to teach effectively (60.8% vs 39.2%, P < 0.001), made the learning environment more comfortable (54.4% vs. 45.6%, P = 0.008) and positively impacted their learning experience (64.6% vs 35.4%, P < 0.001). Students strongly disagreed, disagreed or were neutral when asked if the presence of residents prevented them from taking advantage of patient care opportunities (68.4% vs. 31.6%, P = 0.014). All of these results were statistically significant.

**Table 1.** Student survey responses.

Question	SD, D, N (%)	A, SA (%)	P-value
I generally enjoy working with residents	9 (11.4)	70 (88.6)	<0.001
I feel safer and more willing to ask questions when I am with residents compared to attending physicians	36 (45.6)	43 (54.4)	0.244
Residents are generally willing to teach more than attendings	52 (65.8)	27 (34.2)	0.157
I learn more from residents than attending physicians	51 (64.6)	28 (35.4)	0.042
Residents allow me to become more involved in patient care than attending physicians	44 (55.7)	34 (43)	0.079
Residents allow me to become more involved in physical exams than attending physicians	48 (60.8)	31 (39.2)	0.323
I feel that residents have enough medical knowledge to be effective teachers	11 (13.9)	68 (86.1)	<0.001
The presence of residents in the AOGC clinic enhanced my learning during my outpatient OB/GYN rotation	25 (31.6)	54 (68.4)	<0.001
The residents were able to teach medical students effectively in the AOGC clinic	31 (39.2)	48 (60.8)	<0.001
I would prefer to work directly with attending physicians (not residents) in the outpatient OBGYN clinic setting	48 (60.8)	31 (39.2)	<0.001
The presence of residents in the AOGC clinic prevented me from taking advantage of patient care opportunities	54 (68.4)	25 (31.6)	0.014
The presence of residents in the AOGC clinic made the learning environment more comfortable	36 (45.6)	43 (54.4)	0.008
Residents in the AOGC clinic were too busy to provide instruction	49 (62)	30 (38)	0.976
My learning experience was positively impacted because there were residents in the AOGC clinic	28 (35.4)	51 (64.6)	<0.001

*Abbreviations: SD, strongly disagree; D, disagree; N, neutral; A, agree; SA, strongly agree; AOGC, Academic Obstetrics and Gynecology Clinic which is the equivalent of the OB/GYN resident continuity clinic at SJMH

Results were mixed when students were asked to compare residents to attendings. When asked if they learn more from residents than attending physicians; 28 (35.4%) strongly agreed or agreed, 37 (46.9%) were neutral and 14 (17.7%) disagreed or strongly disagreed. When asked if they would prefer to work directly with attending physicians (not residents) in the outpatient OB/GYN clinic setting; 31(39.2%) strongly agreed or agreed, 30 (38.0%) were neutral and 18 (22.8%) disagreed or strongly disagreed.

Subgroup analyses included age, gender and whether they were medical or physician assistant students. For age, the cutoff of 27 was used as a proxy for other life experiences. Younger (< 27 years) students were more likely to prefer working directly with attendings than older (≥ 27 years) students, (Likert scale mean 3.7 vs 3.0, P = 0.003). Medical students were more likely to prefer working directly with attendings than physician assistant students (Likert scale mean 3.55 vs 2.67, P = 0.003).

### Resident survey

Of the 24 surveys sent out, 21 residents (87.5%) responded. This included 14 residents with more than two years of experience and 7 junior residents with less than two years of experience. A little over half of respondents (52.4%) agreed or strongly agreed that they enjoyed working with students, which was not statistically significant (P = 0.183). However, most respondents who did not answer ‘agree’ or ‘strongly agree’ were ‘neutral’, and the typical score was significantly greater than ‘disagree’ (85.7% neutral, agree, or strongly agree, 14.3% disagreed or strongly disagreed, P < 0.001). The majority of residents felt that teaching was an important part of their job (81.0% vs 19.0%, P < 0.001) and no one strongly disagreed or disagreed with this statement.

As shown in Table 2, the majority of residents agreed or strongly agreed that they were too busy with clinical demands to teach (61.9% vs. 38.1%, P = 0.04) and felt less efficient with patient care due to the demands of teaching (71.4% vs 28.5%, P < 0.001). Residents strongly disagreed, disagreed or were neutral when asked if medical students decreased their overall workload (90.5% vs 9.52%, P < 0.001) or had a negative effect on the relationship they had with their patients (95.2% vs. 4.76%, P = 0.002). We also performed statistical analyses comparing age and level of training amongst resident respondents and found no statistically significant differences.

**Table 2.** Resident survey responses.

Question	SD, D, N (%)	A, SA (%)	P-value
I enjoy working with medical students in the AOGC clinic	10 (47.6)	11 (52.4)	0.183
Overall, I feel that having medical students in the AOGC clinic positively impacts patient care	12 (57.1)	9 (42.9)	0.461
I consider teaching medical students an important part of my job	4 (19)	17 (81)	< 0.001
I feel my learning suffers (I learn less) due to the presence of medical students in the AOGC clinic	14 (66.7)	7 (33.3)	0.568
I am able to teach students effectively in the AOGC clinic	18 (85.7)	3 (14.3)	0.065
I am too busy with clinical demands to teach medical students in AOGC clinic	8 (38.1)	13 (61.9)	0.04
The presence of students in the AOGC clinic has a negative effect on the relationship I have with my patients	20 (95.2)	1 (4.76)	0.002
I feel that I am less efficient with patient care due to the demands of teaching medical students in the AOGC clinic	6 (28.5)	15 (71.4)	< 0.001
Medical students in the AOGC clinic help augment my learning (I learn more)	11 (52.4)	9 (42.9)	0.66
I feel that medical students improve my efficiency and decrease my overall workload in the AOGC clinic	19 (90.5)	2 (9.52)	0.001

*Abbreviations: SD, strongly disagree; D, disagree; N, neutral; A, agree; SA, strongly agree; AOGC, Academic Obstetrics and Gynecology Clinic which is the equivalent of the OB/GYN resident continuity clinic at SJMH

Although not statistically significant, residents did express concerns about the presence of students in their continuity clinic. One third of residents agreed or strongly agreed that their learning suffered due to the presence of students (33.3% vs 66.7%, P = 0.568) and few residents agreed or strongly agreed that they were effective teachers (14.3% vs. 85.7%, P = 0.065).

## Discussion

Based on our survey responses, both residents and students have overall positive views regarding their interactions in RCC. Students felt residents were effective teachers in this setting and made the learning environment more comfortable. Residents considered teaching an important part of their job but expressed concern that they were too busy to teach and their clinical efficiency suffered. Our findings are consistent with the previous Harvard Medical School study addressing student preferences for preceptors in ambulatory OB/GYN [16]. Students recognized both residents and attending physicians contribute to their learning and did not necessarily prefer to work with attendings directly.

Many residents worried about their ability to balance the responsibilities of both clinical care and teaching. This highlights the importance of developing efficient and focused resident teaching skills. Formal training and mentoring for the resident-as-teacher have also been shown to improve resident satisfaction and overall job performance [17–19]. Potential target areas specific to this fast-paced ambulatory setting include enhancing the use of validated tools such as the One Minute Preceptor (OMP) [20]. Residents can also be coached to improve their patient care efficiency by incorporating student education into patient counseling and proactively delegating chart preparation, note writing, and literature reviews. Other areas for resident support include active teaching from the faculty level, which is imperative to both resident and student education [21].

The limitations of our study include a small sample size, especially in our resident cohort, and this may have limited our ability to detect statistically significant differences between sample subgroups. The student and resident surveys were also conducted at a single institution, which limits the generalizability of our results. Further research should focus on how residents and students can best work and learn together in a symbiotic and mutually beneficial manner.

The initial purpose of this study was to determine if students rotating in OB/GYN at SJMH should be placed directly with attendings for their ambulatory experience or continue to rotate in the RCC. These results demonstrate that continuing to have students rotate in RCCs is worthwhile and fosters a positive learning environment for both students and residents.

## Conclusions

The results of our study demonstrate that both residents and students have positive views regarding their interactions with one another in an RCC. Students perceived residents to be effective teachers. However, residents worried about their ability to balance the responsibilities of both clinical care and teaching.
